# Relationship between Immune Parameters and Organ Involvement in Children with Henoch-Schonlein Purpura

**DOI:** 10.1371/journal.pone.0115261

**Published:** 2014-12-16

**Authors:** Yan-xiang Pan, Qing Ye, Wen-xia Shao, Shi-qiang Shang, Jian-hua Mao, Ting Zhang, Hong-qiang Shen, Ning Zhao

**Affiliations:** 1 Clinical Laboratory, The Children’s Hospital of Zhejiang University School of Medicine, Hangzhou, PR China; 2 Clinical Laboratory, Hangzhou First People’s Hospital, Hangzhou, PR China; 3 Zhejiang Chinese Medical University, Hangzhou, PR China; 4 Zhejiang Key Laboratory for Diagnosis and Treatment of Neonatal Diseases, Hangzhou, PR China; 5 The Nephrology Department, The Children’s Hospital of Zhejiang University School of Medicine, Hangzhou, PR China; Centers for Disease Control and Prevention, United States of America

## Abstract

Henoch-Schonlein purpura (HSP) is the most common type of connective tissue diseases which increasingly occurs in children in recent years and its pathogenesis remains unclear. In order to explore the immune parameters and underlying pathogenesis mechanism of children with HSP, the study involved 1232 patients with HSP having different clinical symptoms and their laboratory indicators were evaluated. Th1/Th2 imbalance and overactivity of Th2 cells can cause increase in the synthesis and release of immunoglobulins in children with HSP. The number of red blood cells and white blood cells in urine was directly proportional to the level of IgA and inversely proportional to the level of serum complements (C3 and C4). Activation of these complements caused by immunoglobulin in patients with HSP plays an important role in renal injury. The urinary protein content in children with HSP along with proteinuria was positively correlated with IgE level, and IgE mediated type 1 hypersensitivity can cause increase in capillary permeability and weakened the charge barrier; hence, it could be considered as one of the causes of proteinuria in HSP. Additionally, the NK cells percentage was reduced and impaired immune function of NK cells were related to the immune injury of the digestive tract and kidney.

## Introduction

Henoch-Schonlein purpura (HSP) is the most common type of connective tissue diseases and is a kind of systemic small vessel vasculitis. A large number of recent reports showed an apparent increase in the incidence of children with HSP [1]–[3]. The main clinical manifestation of HSP is purpura without thrombocytopenia characterized by bilateral symmetric distribution of double lower limbs, palpable purpuric rash, abdominal pain, arthralgia, bloody stool, hematuria, and/or proteinuria [4], [5].

However, the exact pathogenesis of HSP remains unknown [6]–[8]. The present study measured the immunoglobulins IgG, IgM, IgA, IgE, complements C3 and C4, T lymphocyte subgroup, B cells, NK cells and C-reactive protein (CRP) in the acute phase of HSP and compared with patients with HSP with different clinical symptoms; hence, this study aimed to find a connection between cellular, humoral immunity, and HSP pathogenesis.

## Materials and Methods

### Objects of study

This was a prospective observational study proceeding from January 2010 to December 2013. The research was approved by ethics committee of zhejiang university medical school affiliated children's hospital. Parents or guardians signed written informed consent for all minors involved in the study. Children met the following criteria were included in this study: (1) children are younger than 18 years; (2) children have been diagnosed as HSP by a doctor according to the standard diagnostic criteria, EULAR/PRINTO/PRES criteria for Henoch-Schonlein purpura [9]; (3) Patients with any other pre-existing disease were excluded from the study. Four hundred healthy children were randomly selected as the normal control. In the acute phase of HSP, five tubes of blood samples were immediately collected for serum Th1/Th2 cytokine, complement, immunoglobulin, T lymphocyte subsets, B cells, NK cells and CRP determination.

The definition of proteinuria: urinary protein is greater than 150 mg/24 h. The definition of haematuria: the red blood cell number is greater than 5 per high magnification of microscopes. The definition of hemafecia: stool occult blood test is positive after rule out the influence of drugs and food.

### Cytokines, T cell subsets and IgE levels in serum

Please refer to the previous papers for the details of the cytokine detection method [10]–[11]. Briefly, the blood samples were centrifuged at 1,000 g for 20 min at 20°C after clotting. The pellets were discarded and the serum was collected carefully. The amount of Th1 and Th2 cytokine in serum was assessed by 320 flow cytometry immediately. The concentration of IL-2, IL-4, IL-6, IL-10, tumor necrosis factor (TNF)-a, and interferon (IFN)-γ were quantitatively determined by the CBA kit–BDTM CBA Human Th1/Th2 Cytokine Kit II (BD Biosciences, San Jose, CA). The standard curve was set up for each individual set of reagents. The minimal and maximum detection limits for all six cytokines were 1.0 and 5,000 pg/mL, respectively.

T cell subsets were detected by multicolor flow cytometry (FAcs calibur, BD, USA) using blood samples with heparin anticoagulant. The mouse anti-human monoclonal antibodies of CD3-FITC, CD4-APC, and CD8-PE, and other reagents used in the study were all purchased from BD Company (USA). B cell and NK cell were detected in the same way using monoclonal antibodies of CD20, CD3, CD16 and CD56. The percentage and significance of test results were analyzed by MultiTEST software. The total IgE level of the serum was detected by the total IgE kit provided by Pharmacia Companies in the United States.

### Immunoglobulin, complement and CRP level detection

Immunoglobulin and complement were detected by Specific protein analyzer (SIEMENS BN-II, Germany SIEMENS Company). Concentrations of CRP were measured by the QuikRead go equipment with QuikRead go CRP kits.

### Detection of urine protein, urine white blood cell and urine red blood cell

Urinary protein was measured using Roche Modular P800 biochemical analyzer (Germany). The number of red blood cell and white blood cell in urine was quantified by SYSMEX UF-1000 automatic urinary sediment analyzer (Japan).

### Statistical analysis

The comparisons between the two groups were performed using the χ^2^ or Fisher’s exact test for categorical variables and Mann–Whitney U test for continuous variables. The Pearson correlation was used for correlation analysis between the two variables. Above statistical analyses was performed using SPSS Statistics18.0 software. *P*<0.05 was considered to be statistically significant.

## Results

### Characteristics of patients

This prospective study included 1232 patients with HSP who were admitted at the nephrology department, Children’s Hospital of Zhejiang University School of Medicine, hangzhou, China. During January 2010 to December 2013, patients with mean age of 5.24 years (range, 1 to 14.6 years) were involved in this study. The gender composition was 700 boys and 532 girls, suggesting that the boys may have a higher probability of HSP than girls (P<0.05). However, there were no statistical difference in gender and age (P = 0.54) between patients with HSP and 400 healthy children (as normal control). 80.03% of patients with HSP had a recent history of upper respiratory tract infection (URI) since 1 to 3 weeks.

**Figure 1:**
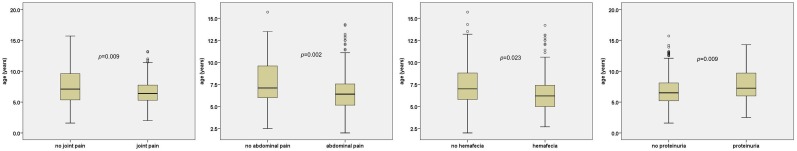
Age distribution of patients with HSP with different clinical symptoms.

The age distribution of patients with HSP with/without joint pain was 6.8 years (range, 1.6 to 15.7 years) and 5.6 years (range, 2.5 to 7.5 years), respectively which showed a significant difference (Z = −3.603, P = 0.009). Patients with HSP with/without abdominal pain were 6.4 years (range, 2 to 14.3 years) and 7.1 years (range, 2.5 to 15.7 years) old, respectively which showed a significant difference (Z = −3.053, P = 0.002). Patients with HSP with/without hemafecia were 6.2 years (range, 2.7 to 14.2 years) and 7 years (range, 2 to 15.7 years) old, respectively which showed a significant difference (Z = −2.268, P = 0.023). Patients with HSP with/without proteinuria were 7.25 years (range, 2.5 to 14.3 years) and 6.5 years (range, 1.6→15.7 years) old, respectively which showed a significant difference (Z = −2.597, P = 0.009). However, hematuresis occurrence in patients with HSP was not associated with age (P>0.05). There was no significant difference between boys and girls on the clinical manifestation (Table 1 and Fig. 1) of HSP.

**Table 1:** The levels of cytokine, T lymphocytes subset, B cell, NK cell, immunoglobulin, complement and CRP from HSP group and normal control group [Median (range)]**.**

Parameters	HSP group (n = 1232)	normal control group (n = 400)	*P value*
Age (year)	5.24(1.0→14.6)	5.75(1.6→15.7)	0.54
IL-2 (pg/mL)	2.2(1.0→17.4)	5.5(2.7→7.8)	<0.01
IL-4 (pg/mL)	3.2(0.9→5.9)	2.8(1.1→4.0)	0.03
IL-6 (pg/mL)	3.6(1.0→1634.5)	4.0(2.4→8.5)	0.62
IL-10 (pg/mL)	2.6(1.0→80.6)	2.4(1.3→7.2)	0.40
TNF-α (pg/mL)	2.3(1.0→59.2)	2.3(1.0→3.0)	0.31
INF-γ (pg/mL)	5.1(1.0→89.9)	4.9(3.8→7.8)	0.36
IgG (g/L)	10.7(2.6→21.5)	8.8(6.7→13.8)	<0.01
IgA (g/L)	2.0(0.6→7.5)	1.1(0.6→2.1)	<0.01
IgM (g/L)	1.2(0.2→4.8)	1.1(0.4→1.5)	0.02
C3 (g/L)	1.2(0.3→2.2)	1.3(0.2→1.5)	0.12
C4 (g/L)	0.3(0.1→0.8)	0.2(0.1→0.4)	<0.01
IgE (IU/mL)	69.1(4.3→3240.0)	27.7(4.3→62.3)	<0.01
CRP (mg/L)	7.0(1.0→64.0)	2.1(1.0→8.0)	<0.01
CD3^+^ (% of positivity)	60.3(7.9→84.6)	66.5(58.3→72.7)	<0.01
CD4^+^ (% of positivity)	29.3(10.5→51.1)	34.8(29.9→47.3)	<0.01
CD8^+^ (% of positivity)	22.6(12.1→27.0)	22.5(16.3→32.1)	0.46
CD4^+^/CD8^+^	1.3(0.4→3.5)	1.6(1.0→2.9)	<0.01
CD20^+^ (% of positivity)	23.0(6.4→51.9)	17.0(15.3→19.7)	<0.01
CD3−CD16+ CD56+ (% of positivity)	7.7(2.1→22.5)	17.2(13.5→23.1)	<0.01

### Clinical symptoms of children with HSP

The most common clinical manifestations in children with HSP were as follows: skin rashes (100%), abdominal pain (59.98%), joint pain (56.01%), hemafecia (34.01%), hematuresis (24.03%), and proteinuria (18.02%) (Table 2). However, 35.71% of children with HSP had three clinical symptoms at the same time; whereas, 38.63% of children with HSP had two clinical symptoms. The remaining patients with HSP had more than three kinds of the above clinical symptoms (Table 2).

**Table 2:** The characteristic of clinical symptoms in HSP children.

Characteristic	Number (percentage)
**Clinical manifestation**	
skin rash	1232 (100.00%)
Abdominal pain	739 (59.98%)
Joint pain	690 (56.01%)
hemafecia	419 (34.01%)
hematuresis	296 (24.03%)
proteinuria	222 (180.02%)
**The number of clinical symptoms**	
2 kinds	476 (38.63%)
3 kinds	440 (35.71%)
4 kinds	224 (18.18%)
5 kinds	76 (6.17%)
6 kinds	16 (1.31%)

### T cell subsets, B cells, NK cells, serum cytokines, immunoglobulins, complements, and CRP levels in the acute phase of HSP

The proportions of CD3+ and CD4+ cells in patients with HSP were significantly lower than healthy children, leading to a significant reduction in the ratio of CD4+/CD8+ (P<0.01). Interleukin-2 (IL-2) levels in children with HSP decreased significantly (P<0.01); whereas, interleukin-4 (IL-4) level increased significantly (P = 0.03). The proportion of CD20+ cells (P<0.01) and the levels of IgE, IgG, IgA, and IgM in children with HSP were higher than healthy children; whereas, the proportions of CD3−CD16+CD56+ cells were lower. In addition to this, the levels of CRP and complement C4 (P<0.01) were significantly higher in patients with HSP than healthy children (Table 1).

### T cell subsets, serum cytokines, immunoglobulins, complements, and CRP levels in children with HSP had different clinical symptoms

**Figure 2:**
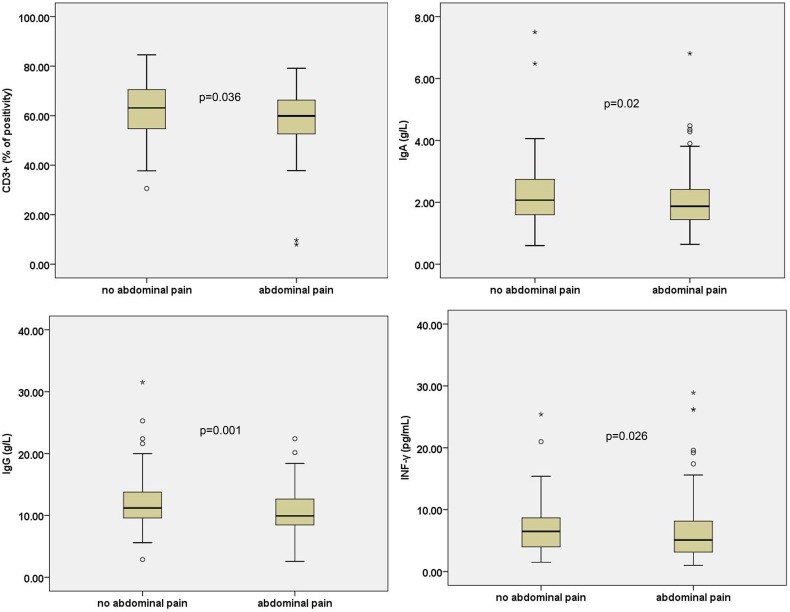
T cell subsets, serum cytokines, and immunoglobulin levels in children with HSP with abdominal pain. All these indicators are statistically significant P<0.05.

**Figure 3:**
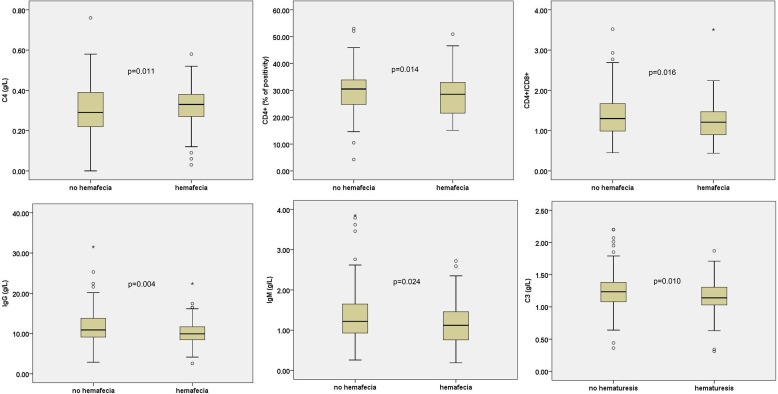
T cell subsets, immunoglobulin, and complement levels in children with HSP with hemafecia or hematuresis. All these indicators are statistically significant P<0.05.

Children with HSP with abdominal pain had lower IgA (Z = −2.326, P = 0.02), IgG (Z = −3.251, P = 0.001), INF-γ (Z = −2.222, P = 0.026), and CD3+ T cells (Z = −2.099, P = 0.036) than children without abdominal pain (Fig. 2). Children with HSP with hemafecia had lower IgG (Z = −2.862, P = 0.004), IgM (Z = −2.26, P = 0.024), CD4+ cells (Z = −2.467, P = 0.014), and ratio of CD4+/CD8+ (Z = −2.416, P = 0.016) than children without hemafecia (Fig. 3).

### The proportion of CD3−CD16+CD56+ and CD20+ cells in patients with HSP with gastrointestinal or kidney injury

**Figure 4:**
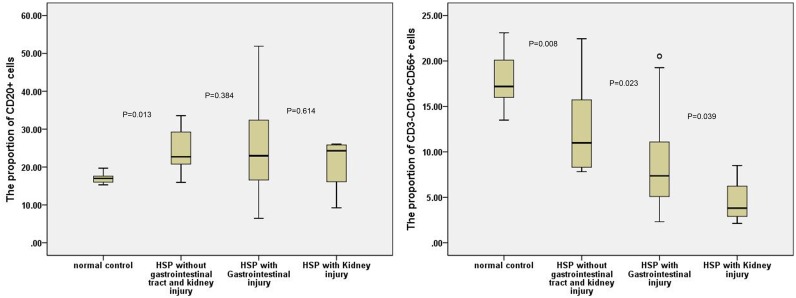
The proportion of CD3−CD16+CD56+ and CD20+ cells in patients with HSP with gastrointestinal or kidney injury.

The results showed that percentage of B cells (CD20+) and NK cells (CD3−CD16+CD56+) increased and decreased (P<0.01), respectively among children with HSP as compared with healthy children. However, the children who were diagnosed with HSP were affected either with digestive tract or kidney injury or not, and their B cells (CD20+) percentage did not increase significantly (P>0.05). However, HSP children with digestive tract injury or kidney injury, NK cells percentage was lower. Additionally, NK cells percentage was lower (P = 0.039) in HSP children with kidney injury than those with digestive tract injury (Fig. 4).

### Correlation between immune indexes and leucocyturia; hematuria and proteinuria

Correlation analysis showed that the number of red blood cells (RBCs) in urine was positively correlated with IgA level in children with HSP having hematuresis (r = 0.288, P = 0.023) and negatively correlated with the proportion of CD4+ T cells (r = −0.253, P = 0.046) and complement C4 level (r = −0.269, P = 0.034). The urinary protein content in children with HSP along with proteinuria was negatively correlated with IL-2 level (r = −0.317, P = 0.023) and positively correlated with IgE level (r = 0.297, P = 0.042). The number of white blood cells was positively correlated with IgA level (r = 0.260, P = 0.003) and negatively correlated with the proportion of CD4+ T cells (r = −0.298, P = 0.001) in children with HSP along with nephritis.

## Discussion

HSP is the most common type of connective tissue diseases and its pathogenesis remains unknown. Studies have shown that it may be correlated with allergy caused by infections, pollen, food, drugs, and/or other factors [12]–[14]. The study found that more than 80% of children with HSP had a recent history of URI since 1 to 3 weeks. This suggested that the prodromic infection with URI may be considered as a major causing factor of HSP. Statistical analysis also indicated that the average level of CRP and C4 are two important inflammatory markers [15] increased in children with HSP; Hence, HSP is in the state of inflammation.

T lymphocyte cells are divided into CD4+ and CD8+ T cells per different surface antigens. It can also be divided into helper (Th), suppressor (Ts), and cytotoxic T cells (Tc) per different immune function [16]. The surface of Th has CD4+ molecules, whereas CD8+ molecules are found on the surface of Ts and Tc [17]. There is a certain balance between CD4+ and CD8+ cells under normal physical circumstances [18]. The results have suggested that the proportion of CD4+ is reduced in children with HSP, resulting in a decline in the ratio of CD4+/CD8+ cells as compared with healthy children. Hence, there is an imbalance between CD4+ and CD8+ T cells, and further changes in Th cell subsets and their roles in the pathogenesis of HSP to be investigated. According to the secretion of cytokines, Th cells can be divided into two subsets: Th1 and Th2, this study mainly focused on balance between Th1 and Th2 cells. [19]. Therefore, the cytokines levels in serum from the children with HSP and healthy children were tested and analyzed, respectively. The results showed that the level of IL-2, proportions of CD3+, and CD4+ cells were reduced in patients with HSP. IL-2 is also called T cell growth factor and is essential for T cell proliferation and differentiation [20]–[23]. This explains that decrease of T cells is accompanied with the decrease of IL-2. In addition, cytokines secreted by Th2 cells such as IL-4 increased significantly in children with HSP and the ratio of IL-4/INF-γ was higher than healthy children. INF-γ is a kind of cytokines secreted by Th1 cells. Besbas N et al suggested that the children with HSP were characterized by Th1/Th2 imbalance and excessive activation of Th2 cells [24]. Theoretically, excessive activation of Th2 cells increases the secretion of cytokines, which could be beneficial for B cell proliferation and differentiation. Then, these increased cytokines could induce B cells to be transformed into plasma cells, allowing increased synthesis and release of immunoglobulin, and causes humoral immune abnormalities [25]. To confirm this hypothesis, the proportions of CD20+ cells and immunoglobulin levels of children with HSP were detected. CD20+ is the specific surface differentiation antigen of B cell, often as a sign of B cell specificity. Our results showed that the number of B cells increased significantly, meanwhile immunoglobulins from the patients with HSP, such as IgG, IgM, IgA and IgE, also increased significantly than healthy children. This finding is consistent with the research by Davin et al. [26]. Although the exact pathogenesis of HSP remains unknown, it is considered to be a small vessel leukocytoclastic vasculitis mediated by an immune complex, which is characterized by the presence of IgA, and it is suggested that unknown antigens stimulate IgA production, activating some pathways that leads to vasculitis [27]–[31]. Deposition of the IgA in immune complexes is well boosted by subsequent increase in local capillary permeability [26].

The results showed that although percentage of B cells increased (P<0.01) among children with HSP as compared with healthy children. However, the children who were diagnosed with HSP were affected either with digestive tract or kidney injury or not, and their B cells percentage did not exist significant difference (p>0.05). Thus it is believed that the rising percentage of B cell is not determinant of digestive tract injury and kidney injury. The results also showed that NK cells (CD3−CD16+CD56+) percentage decreased (P<0.01) among children with HSP compared with healthy children. In HSP children with digestive tract injury or kidney injury, NK cells percentage was lower. Additionally, NK cells percentage was lower (P = 0.039) when HSP children with kidney injury than those with digestive tract injury. This suggests that NK cells play an important role in the digestive tract and kidney injury, especially in the kidney injury. NK cells have the ability to remove foreign antigens in the body; thereby reducing the number of foreign antigen accumulation in the capillaries, especially in the areas with rich capillaries such as digestive tract and kidneys. Accumulation of foreign antigen causes strong immune response and results in the immune injury of digestive tract and kidneys. In addition, IL-2 was found at a low level in children with HSP, which could reduce NK cells’ immune function; therefore, it could affect the ability to remove foreign antigens and increase injury of the digestive tract and kidney. Hence, the NK cells percentage reduction and impaired immune function are related to the immune injury of the digestive tract and kidney in patients with HSP.

The abdominal pain and bloody stool account for 59.98% and 34.01% in patients with HSP, respectively. Statistical analysis showed lower serum immunoglobulins, Th cells and CD4+/CD8+; and higher complement levels in patients with HSP with the above clinical symptoms as compared those without the above clinical symptoms. This suggests that besides the rising percentage of B cell, the gastrointestinal tract involvement is related to CD4+ and CD8+ T cells imbalance. The proportions of HSP patients with hematuria and proteinuria were 24.03% and 18.02%, respectively. The results have showed the patients with HSP with renal injury had higher serum immunoglobulin levels and lower complements as compared those without hematuria, proteinuria, and normal control. The numbers of RBCs and WBCs in urine are directly proportional to the level of IgA and inversely proportional to the level of serum complements. These data were suggested that complements activation may cause by immunoglobulin in patients with HSP plays an important role in renal injury. The urinary protein content in children with HSP with proteinuria was positively correlated with IgE level (r = 0.297, P = 0.042). This suggests that the IgE mediated type 1 hypersensitivity causes increase in capillary permeability and weakens the charge barrier; hence, it is one of the causes of proteinuria in patients with HSP.

In additional, younger patients had more joint and abdominal complaints, however older patients had more renal involvement. Although the specific mechanism is not clear now, but age is believed to be a risk factors for renal involvement [32]. In conclusion, HSP in older patients had a higher risk of progression to renal involvement, then more aggressive treatment and extended follow-up with repeated urinalysis are often necessary in older HSP patients despite initially normal renal findings.
